# Extracting quasi-steady Lagrangian transport patterns from the ocean circulation: An application to the Gulf of Mexico

**DOI:** 10.1038/s41598-018-23121-y

**Published:** 2018-03-26

**Authors:** R. Duran, F. J. Beron-Vera, M. J. Olascoaga

**Affiliations:** 1 College of Earth Ocean and Atmospheric Sciences, Oregon State University, Corvallis, Oregon, USA; 2 Department of Atmospheric Sciences, Rosenstiel School of Marine and Atmospheric Science, University of Miami, Miami, Florida USA; 3 Department of Ocean Sciences, Rosenstiel School of Marine and Atmospheric Science, University of Miami, Miami, Florida USA; 4 Present Address: Theiss Research, La Jolla, CA 92037 USA

## Abstract

We construct a climatology of Lagrangian coherent structures (LCSs)—the concealed skeleton that shapes transport—with a twelve-year-long data-assimilative simulation of the sea-surface circulation in the Gulf of Mexico (GoM). Computed as time-mean Cauchy–Green strain tensorlines of the climatological velocity, the climatological LCSs (cLCSs) unveil recurrent Lagrangian circulation patterns. The cLCSs strongly constrain the ensemble-mean Lagrangian circulation of the instantaneous model velocity, showing that a climatological velocity can preserve meaningful transport information. The quasi-steady transport patterns revealed by the cLCSs agree well with aspects of the GoM circulation described in several previous observational and numerical studies. For example, the cLCSs identify regions of persistent isolation, and suggest that coastal regions previously identified as high-risk for pollution impact are regions of maximal attraction. We also show that cLCSs are remarkably accurate at identifying transport patterns observed during the Deepwater Horizon and Ixtoc oil spills, and during the Grand LAgrangian Deployment (GLAD) experiment. Thus it is shown that computing cLCSs is an efficient and meaningful way of synthesizing vast amounts of Lagrangian information. The cLCS method confirms previous GoM studies, and contributes to our understanding by revealing the persistent nature of the dynamics and kinematics treated therein.

## Introduction

Lagrangian transport is a difficult oceanographic problem for which solutions are frequently needed. Sensitivity to initial conditions or to the precision of the velocity field require more attention to detail than what we are usually able to afford. Even if those details could be resolved, making progress with only a few general guidelines would be ideal, appraising many new applications as practical. For example, those tasked with planning for environmental-pollution response and prevention would like to have information applicable to generic oil spills. Useful information includes identification of recurrent trajectory patterns at the sea surface, identification of regions with a higher risk of contamination, and identification of isolated areas, where pollution is unlikely to enter or leave. This provides motivation for revealing structures that evolve slowly relative to a typical Lagrangian timescale, yet tend to organize transport. In particular, we are interested in structures that persist for periods much longer than the Lagrangian-velocity decorrelation time, which is order one day at the sea surface, and is a basic indicator of Lagrangian predictability^1^. With the above in mind, we analyze a long record of surface currents in the Gulf of Mexico (GoM) from a data-assimilative simulation using tools from the theory of nonlinear dynamical systems. These tools enable objective (i.e. observer-independent) identification of key material lines that organize Lagrangian transport, often referred to as Lagrangian coherent structures (LCSs)^2^. Samelson^3^ reviews the original heuristic approaches to LCSs and includes a fluid-dynamical account of the terminology; Haller^4^ reviews the recent, rigorous LCS theory employed here.

We show that it is possible to construct a climatology of LCSs that strongly constrains the ensemble-mean Lagrangian circulation that is advected by the instantaneous ocean-model velocity. The quasi-steady transport patterns revealed by the climatological LCSs also agree well with aspects of the GoM Lagrangian circulation that have been described in several previous studies. For instance, by identifying regions of persistent isolation or by suggesting that the coastal regions that have been reported as high-risk for pollution impact are regions of maximal attraction. Finally, we also show that climatological LCSs agree remarkably well with surface-drifter and oil-spill distributions and beachings.

## Methods

### Lagrangian coherent structures

The LCSs are material lines (i.e. composed at all time of the same fluid particles) that strongly influence nearby fluid over a given time interval. The class of influence can vary, but it always produces an organized transport pattern for which the underlying LCS serves as a theoretical backbone. Organized patterns in the distribution of tracers are often seen in natural flows, it is the underlying LCS that shape these organized patterns, and therefore it is the LCSs that are of interest.

The starting point for LCS detection is the motion equation for fluid particles, given by the following two-dimensional nonautonomous dynamical system:

1 $$\frac{{\rm{d}}{\bf{x}}}{{\rm{d}}t}={\bf{v}}({\bf{x}},t),$$

where **v**(**x**, *t*) is the velocity of the fluid. By solving this equation for fluid particles at positions **x**_0_ at initial time *t*_0_, one obtains a map that takes the particles to positions **x** at a time *t*_1_, namely, ${{\bf{F}}}_{{t}_{0}}^{{t}_{1}}({{\bf{x}}}_{0})\,:=\,{\bf{x}}({t}_{1};{{\bf{x}}}_{0},{t}_{0})$.

The geodesic LCS theory^5,6^ builds on a notion of strain which is objectively measured by the Cauchy–Green (CG) strain tensor,

2 $${{\bf{C}}}_{{t}_{0}}^{{t}_{1}}({{\bf{x}}}_{0})\,:=\,{[{\rm{D}}{{\bf{F}}}_{{t}_{0}}^{{t}_{1}}({{\bf{x}}}_{0})]}^{{\rm{\top }}}{\rm{D}}{{\bf{F}}}_{{t}_{0}}^{{t}_{1}}({{\bf{x}}}_{0}),$$

where

3 $$\begin{array}{c}{\rm{D}}{{\bf{F}}}_{{t}_{0}}^{{t}_{1}}({{\bf{x}}}_{0})=[\begin{array}{cc}\displaystyle \frac{{\rm{\partial }}x}{{\rm{\partial }}{x}_{0}} & \displaystyle \frac{{\rm{\partial }}x}{{\rm{\partial }}{y}_{0}}\\ \displaystyle \frac{{\rm{\partial }}y}{{\rm{\partial }}{x}_{0}} & \displaystyle \frac{{\rm{\partial }}y}{{\rm{\partial }}{y}_{0}}\end{array}].\end{array}$$

In the above definitions, both *t*_1_ < *t*_0_ or *t*_1_ > *t*_0_ are acceptable. Let 0 < *λ*_1_(**x**_0_) < *λ*_2_(**x**_0_) and **ξ**_1_(**x**_0_) ⊥ **ξ**_2_(**x**_0_) be eigenvalues and normalized eigenvectors of (2). A local normal-growth measure of the unit normal, **n**_0_, along a material line at time *t*_0_ is given by^7^:

4 $${\rho }_{{t}_{0}}^{{t}_{1}}({{\bf{x}}}_{0},{{\bf{n}}}_{0})\,:=\,\frac{1}{\sqrt{{{\bf{n}}}_{0}\cdot {{\bf{C}}}_{{t}_{0}}^{{t}_{1}}{({{\bf{x}}}_{0})}^{-1}{{\bf{n}}}_{0}}}.$$

Of particular interest for our purposes are attracting LCSs, as these delineate pathways for Lagrangian transport. An LCS that attracts nearby particle trajectories over a finite-time interval [*t*_1_, *t*_0_], where *t*_1_ = *t*_0_ + *T* and *T* < 0, is a squeezing Cauchy–Green strain tensorline or *squeezeline*^8^, i.e., a curve $s\mapsto {\bf{x}}(s)$ which satisfies^5,6^

5 $$\frac{{\rm{d}}{\bf{x}}}{{\rm{d}}s}={{\boldsymbol{\xi }}}_{1}({\bf{x}})$$

and^9^

6 $${\rho }_{{t}_{0}}^{{t}_{1}}({\bf{x}})=\sqrt{{\lambda }_{2}({\bf{x}})}\, > \,1.$$

We note that different time intervals define different finite-time dynamical systems. If the velocity is non-divergent, then (6) is guaranteed to be satisfied. The most attracting LCSs in forward time are those with the largest back-in-time normal repulsion ${\rho }_{{t}_{0}}^{{t}_{1}}({\bf{x}})$. When the flow is nondivergent, forward-in-time normal attraction implies tangential stretching. For simplicity we no longer write *ρ*’s dependence on *t*_0_, *t*_1_ and **x** below.

### Climatological Lagrangian coherent structures

Our approach to revealing quasi-steady structures that organize transport consists of two steps. The first is computing a velocity climatology. This step is effectively a low-pass filter for the velocity; it therefore raises the question of how relevant is the climatological velocity for Lagrangian transport applications. We address this question in the first section of the results. The second step consists of averaging several CG tensors, and using this time-averaged tensor to compute LCSs.

In general, computing LCSs requires two choices that may be subjective, namely, choosing the values for *T* and *t*_0_. Towards constructing an objective method to extract quasi-steady transport patterns, our approach for the choice of *T* is to use a timescale that is relevant to several Lagrangian transport problems, and then test our method for sensitivity to the choice of *T*. Our approach for the choice of *t*_0_ is to choose several initial times over a period that is larger than *T*, compute a CG tensor for each of these initial times, and then average the CG tensors. By averaging over *t*_0_, we circumvent the dependence on *t*_0_. We refer to the LCSs computed from this time-averaged CG tensor as climatological LCSs (cLCSs). As we will see below, cLCSs turn out to be effectively equivalent to the superposition of LCSs from the dynamical systems in the averaging period.

In the following subsections we describe the velocity field used in this study and how the climatological velocity is computed. we justify our choice for *T*, and explain the averaging of the CG tensor. We also report numerical aspects of our computations, and results from several sensitivity tests.

### Velocity data and climatology

For the velocity **v**(**x**, *t*) we use 12 years of daily sea-surface velocity from the Hybrid-Coordinate Ocean Model^10^ (HyCOM), forced by the US Navy Operational Global Atmospheric Prediction System. The resolution is about 4 km, appropriate for our search of persistent material deformation shaping global transport. In the Supporting Information (Appendix A), we show that the motions we report are caused by confluence (divergence-free attraction) rather than convergence (attraction with negative divergence).

From the HyCOM-GOM10.04 analysis, experiment 20.1 was used for years 2003 through 2009, experiment 31 for January through March 2010, and experiment 32.5 for April 2010 through year 2014. This 12-year period (2003–2014) was used because HyCOM simulations include the Navy Coupled Ocean Data Assimilation^11,12^ (NCODA).

For simplicity, each data-year is defined to be the first 360 days of the calendar year, and therefore months are composed of 30 days. The only exception is 2003 spanning days 2 to 361 due to availability. Temporal resolution is for the most part a daily instantaneous field except for a 4-day gap in 2004, a 2-day gap in 2009 and a 1.5-day gap in 2014. Cubic interpolation was used to remediate these gaps and keep the time between velocity fields at 24 hours. The first day of the velocity climatology is obtained by averaging the first day of the 2003–2014 time series, and so on.

Regarding the velocity’s spatial and temporal resolution, we note that in the Gulf of Mexico, the kinetic energy spectra as represented by models at resolutions of up to about 1 km are steep enough for bulk Lagrangian calculations to be largely insensitive to fine velocity details in space and time^13,14^. Additionally, Keating *et al*.^15^ showed that interpolation can satisfactorily ameliorate even a very coarse temporal resolution. We also note that ocean models with a higher spatial resolution than the one we use, are currently undesirable for our purposes because they are known to decrease the model’s skill^16^. The loss of skill at the mesoscale, despite assimilating data, is of particular concern^17^. More generally, there is compelling evidence that our ability to simulate submesoscale processes is still in its early stages (e.g. Soufflet *et al*.^18^).

### The timescale *T* and averaging of the CG tensors

As described above, computing LCS requires choosing values for *T* and *t*_0_. A few days to a week is a critical timescale for oil-spill response^19^, search and rescue operations^20,21^, and larval recruitment and algal blooms^22,23^. Thus, we choose *T* = −7 days. Because we are interested in general patterns and not a particular event starting at a particular time, for each month we compute CG tensors with *T* = −7 days fixed, and initial times *t*_0_ ∈ {8, 10, 12,…,30} days (each month consists of 30 days of data). The monthly-mean CG tensor is then the average of the CG tensors from these back-in-time, 7-day flow maps, initiated every other day in that month.

### Numerics and sensitivity tests

All integrations are done with a Runge–Kutta 4(5) pair (i.e. with adaptative time step) and cubic interpolations. The computational domain covers the GoM with a mean grid spacing of 1.7 km; an auxiliary grid of 4 points separated by 0.1 km and centered at each grid point of the main grid is used to evaluate the centered derivatives with which (3) is approximated. The numerical implementation of geodesic LCS detection is documented at length^5,6,8,24^; a software tool is also available^25^.

A welcome finding through sensitivity tests was that our results are robust. Tests using instantaneous velocity fields with six, twelve or twenty-four hour intervals, suggest that different temporal resolutions will not affect the results. Tests using *T* ∈ {−5, −7, −10, −15, −20} days, suggest that our results do not depend sensitively on *T*. We also get the same results when using a second order Runge-Kutta. In the Supporting Information (Appendix B) we show that doubling the resolution of the grid used to compute (3) does not change our results.

### Additional data

In the results section, we compare cLCSs with several transport patterns to show that they are indeed quasi-steady structures organizing Lagrangian transport. Beyond comparisons with trajectory ensembles using the instantaneous model velocity, and with results from previous studies, we also use three data sets of observed transport patterns for direct comparisons. First we use surface oil images during the *Deepwater Horizon* spill (DwH), produced by the NOAA Experimental Marine Pollution Surveillance Reports (EMPSR; http://www.ssd.noaa.gov/PS/MPS/deepwater.html). They delineate surface oil using satellite imagery from active and passive sensors, overflights and *in situ* observations. Further support for our results is provided by surface-drifter trajectory data from the Grand LAgrangian Deployment (GLAD) in the northern GoM^8,26–28^. Finally, we use data from the *Ixtoc* spill (1979–1980). Availability of observations with good temporal and spatial coverage for this event is restricted to data from the Coastal Zone Color Scanner (CZCS, Nimbus-7 satellite) and Landsat Multispectral Scanner (MSS, Landsat 1–5 satellites). These data were acquired and carefully processesed by Sun *et al*.^29^; we also use the beaching locations in their Table 1.

## Results and Discussion

We begin by verifying that the velocity climatology preserves meaningful transport patterns, and that these patterns are depicted by cLCSs. Once a Lagrangian relationship between the instantaneous and climatological velocities has been established, we compare cLCSs with kinematics previously reported in the Gulf of Mexico, and with some high-profile, observed, transport patterns.

### Comparison with the ensemble-mean transport produced by the model

In this section we show that ensemble-mean transport sustained by the instantaneous HyCOM velocity, conforms to cLCSs that are computed with the velocity’s climatology. Consider an arbitrary tracer distribution *f*_0_(**x**_0_) at time *t*_0_. The image of a point ***x***_0_ under the flow at time *t*_1_ = *t*_0_ + *T*, is ${{\bf{x}}}_{{t}_{0}+T}={{\bf{F}}}_{{t}_{0}}^{{t}_{0}+T}({{\bf{x}}}_{0})$. The advected image of a conserved tracer distribution is (e.g., Froyland *et al*.^30^):

7 $${f}_{{t}_{0}+T}({{\bf{x}}}_{{t}_{0}+T})={f}_{0}({{\bf{F}}}_{{t}_{0}+T}^{{t}_{0}}({{\bf{x}}}_{{t}_{0}+T}){\rm{\det }}\,{\rm{D}}{{\bf{F}}}_{{t}_{0}+T}^{{t}_{0}}({{\bf{x}}}_{{t}_{0}+T})$$

(Note that flow maps preserve orientation, and therefore the Jacobian determinant cannot be negative.)

Consider an ensemble of backward trajectories $\{{{\bf{F}}}_{{t}_{0}+T}^{{t}_{0}}({{\bf{x}}}_{{t}_{0}+T})\}$ with different *t*_0_ for each ${{\bf{x}}}_{{t}_{0}+T}$. The corresponding ensemble-mean backward flow map ${\bar{{\bf{F}}}}_{T}^{\,0}({{\bf{x}}}_{T})$, can be used to evaluate (7), producing an ensemble-mean tracer distribution ${\bar{f}}_{T}({{\bf{x}}}_{T})$.

**Figure 1:**
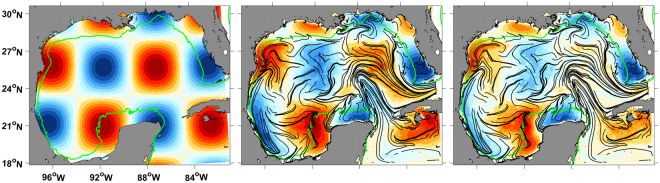
The left panel is the initial distribution (colors, arbitrary units) of a tracer, the 50-m isobath is plotted in green in all panels. The middle panel shows the ensemble-mean distribution resulting from 7-day advections of the initial distribution, under the instantaneous velocity for each January in the climatology, plotted over January’s cLCSs (black lines). The right panel is the same as the middle panel except that the ensemble-mean distribution is the result of 14-day advections. The arbitrary colorscale limits are the same in all panels. Created with Matlab R2016B (www.mathworks.com).

Let *f*_0_(**x**_0_) = $\sin \,\frac{{x}_{0}}{2}\,\sin \,\frac{{y}_{0}}{2}$ with **x**_0_ = 0 roughly at the center of the GoM, conveniently chosen to facilitate the visualization of advection patterns. If we take *t*_0_ over January along each of the 12 years of simulation, the ensemble-mean distributions ${\overline{f}}_{T}({{\bf{x}}}_{T})$ at *T* = 7 and *T* = 14 days show that the instantaneous circulation is strongly constrained by the corresponding January cLCSs (Fig. 1). LCSs are by construction material lines, despite cLCSs not preserving this property, the tracer distribution often stretches along cLCSs, e.g. meridionally along the western GoM, just north of the Yucatan shelf and along the 50-m isobath on the western Yucatan shelf. Also, cLCSs seem to serve as barriers e.g. within the Yucatan shelf around 91°W, separating blue and red tracer. These results do not depend on the choice of month.

### Comparison with known transport patterns

**Figure 2:**
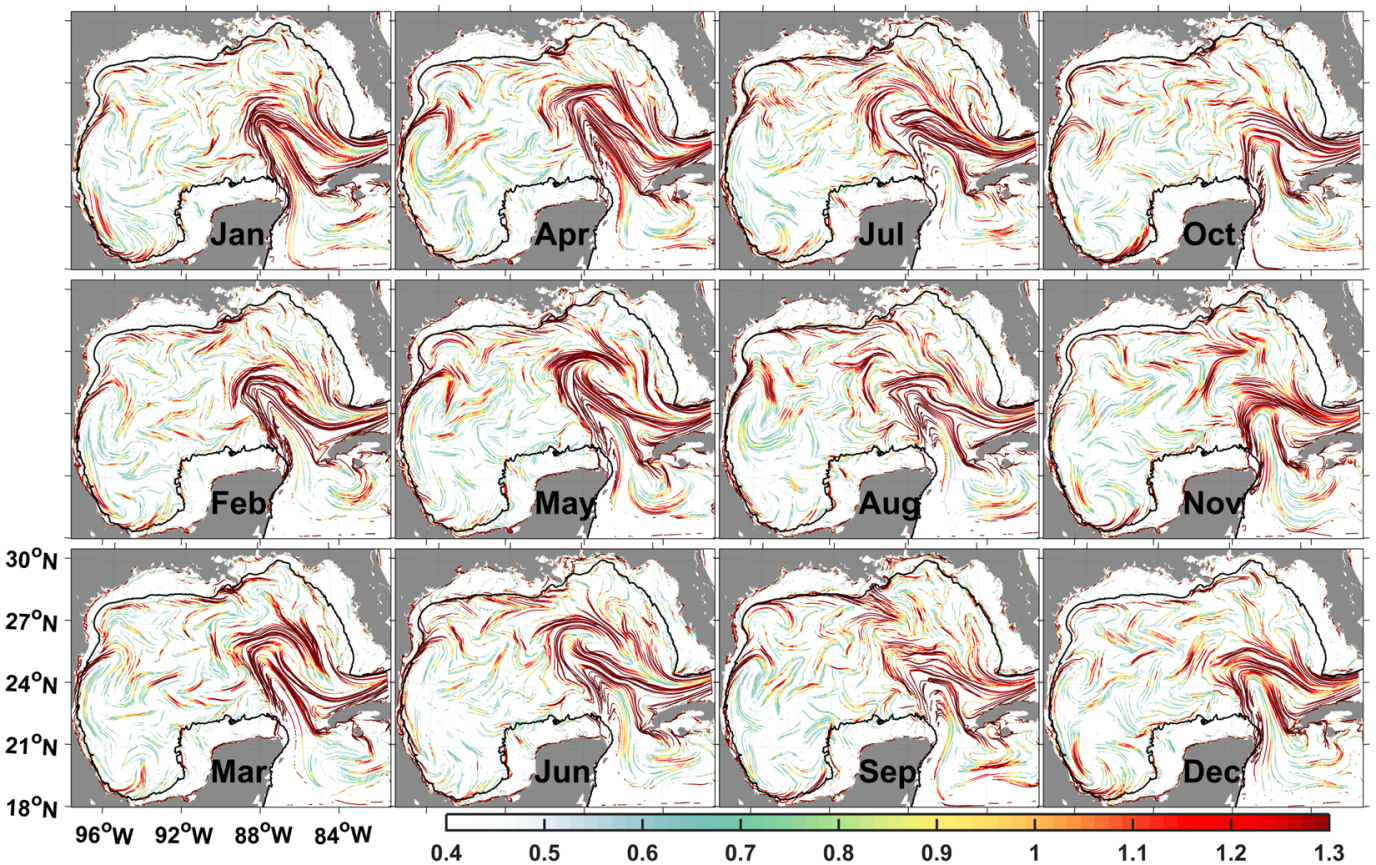
Monthly climatological LCSs (each column is roughly a season) colored according to their climatological attraction strength lnc*ρ*. The 50-m isobath is indicated in black. Created with Matlab R2016B (ww.mathworks.com).

A characterization highlighting important kinematical features in the GoM emerges from the monthly cLCS maps (Fig. 2). We illustrate this through comparisons with known transport patterns and, in a subsection below, with observations.

#### The Loop Current

The Loop Current (LC), a region of persistent attraction, is the predominant feature of the cLCS fields in the GoM interior. In the last three months of the year, the LC does not reach as far north as it does in the previous 9 months. This is consistent with different multi-year studies showing that, in any given year, eddies will most likely have shed by the time the first 9 months of that year are over, cf. Fig. 12 of Vukovich^31^ and Fig. 4 of Lindo-Atichati *et al*.^32^.

#### Western GoM

Between 92–96°W and 19–23°N, climatological attraction c *ρ* is often weak suggesting stagnation relative to the rest of the GoM interior (i.e. deeper than 50 m). However, along the western-boundary 50-m isobath, meridional stretching tends to be relatively strong. Consistently, northward advection along the western margin and retention within the southwestern GoM are the two characteristic dispersion scenarios identified by Zavala-Sanson *et al*.^33^ using drifters released in years 2007–2014, between 93–96°W and 19–20.5°N.

Our climatology often shows offshore transport originating from the western-boundary 50-m isobath around 24–26°N, and from the southwestern 50-m isobath around 19°N and 92–94°W. Enhanced cross-shelf transport in both these regions was identified through satellite data between 1997–2007 by Martínez-López & Zavala-Hidalgo^34^ (cf. their Fig. 7); they propose that both cases of offshore transport are due to convergence of alongshore wind-driven currents, with the western case (24–26°N) modulated by the presence of cyclonic and anticyclonic eddies. Zhang & Hetland^35^ reached a similar conclusion over the Louisiana-Texas (La-Tex) shelf. The study by Gough *et al*.^36^ is, to our knowledge, the first to apply the techniques described in this paper. They confirm offshore transport around 24–26°N by computing cLCSs from an 18-year simulation using the Nucleus for European Modelling of the Ocean (NEMO) model. Their cLCSs agree well with transport from historical drifter observations and synthetic drifters advected with their instantaneous velocity.

#### Coastal risk

Coastal cLCSs with a strong attraction imply an increased risk of environmental impact to the nearby coastline: Lagrangian parcels are persistently attracted to where their trajectories may be subject to effective cross-shelf drivers such as Stokes drift^37,38^.

The vicinity of the Mississippi delta (89.2°W, 29°N) often has an agglomeration of highly-attractive cLCSs; during spring and summer, the shelf just to the east (85–89°W, ~30°N) has many cLCSs with values of c *ρ* that are high relative to other shelves (see also Fig. 4 in the Supplemental Information). This agrees well with transport and beaching of DwH oil, from April through August of 2010^37,38^. It also agrees with several studies using multi-year trajectory simulations to determine the likely outcome for a spill originating at the Macondo well under spring and summer conditions: The vicinity of the Mississippi delta is most-at-risk, followed by the shelf and coast to the east up to about 85°W; the LC is also found to attract trajectories in these multi-year simulations, although oil from the DwH did not reach it^39–41^. These studies use velocity fields from years 1992–2008, 1993–1998 and 1992–2007, respectively, to compute probability of impact based on a point-source oil spill, and forward-in-time integrations of the instantaneous velocity. Thus, while the model we use requires due caution when interpreting coastal circulation, our results seem to confirm these studies, and the first part of a conclusion in Weisberg *et al*.^38^ (who studied DwH oil beaching using different models and observations): “In essence it is found that the circulation gets the oil to the vicinity of the beach, whereas the waves, via Stokes drift, are responsible for the actual beaching of oil.”

Our analysis identifies that the interior of the three wide shelves of the GoM–the West Florida, La-Tex and Yucatan shelves–are isolated throughout the year; the 50-m isobath being a good indicator for the transport barrier. Shallower than this isobath, c *ρ* is for the most part negligible, implying low stirring activity, and that water parcels within the shelves are unlikely to have originated from outside. This is consistent with what is expected from wide shelves^42^. However, some cLCSs with strong c *ρ* values can be seen sporadically within some of these shelves, e.g. in the Yucatan shelf near (91°W, 21°N), specially during the summer. Also, highly-attractive cLCSs may persist near the coastline.

The isolation of the West Florida shelf has been documented from observations and numerical models^43–45^, while observational and numerical studies have also noted that the vicinity of the 50-m isobath separates distinct kinematical and dynamical regimes within that shelf^46–48^. Thus, our results seem a confirmation of the underlying dynamics described in these studies.

The cLCSs reported by Gough *et al*.^36^ accurately identify transport barriers and isolation for the La-Tex shelf, as evaluated through comparisons with synthetic drifters (advected by their instantaneous velocity) and historical satellite-tracked drifters. Thus providing an independent confirmation for isolation within the La-Tex shelf. Isolation for the Yucatan shelf will be illustrated below, with observations from the Ixtoc oil spill.

Highly-attractive cLCSs persist along the western coastline of the GoM, this pattern is consistent with the coastal vulnerability (attraction of synthetic drifters) found by Thyng & Hetland^49^, from about 24 to 29°N (cf. their Figs 3 and 8). And as we will see, it is also consistent with oil beaching from the Ixtoc oil spill.

### Direct comparison with observations

#### The “tiger tail”

**Figure 3:**
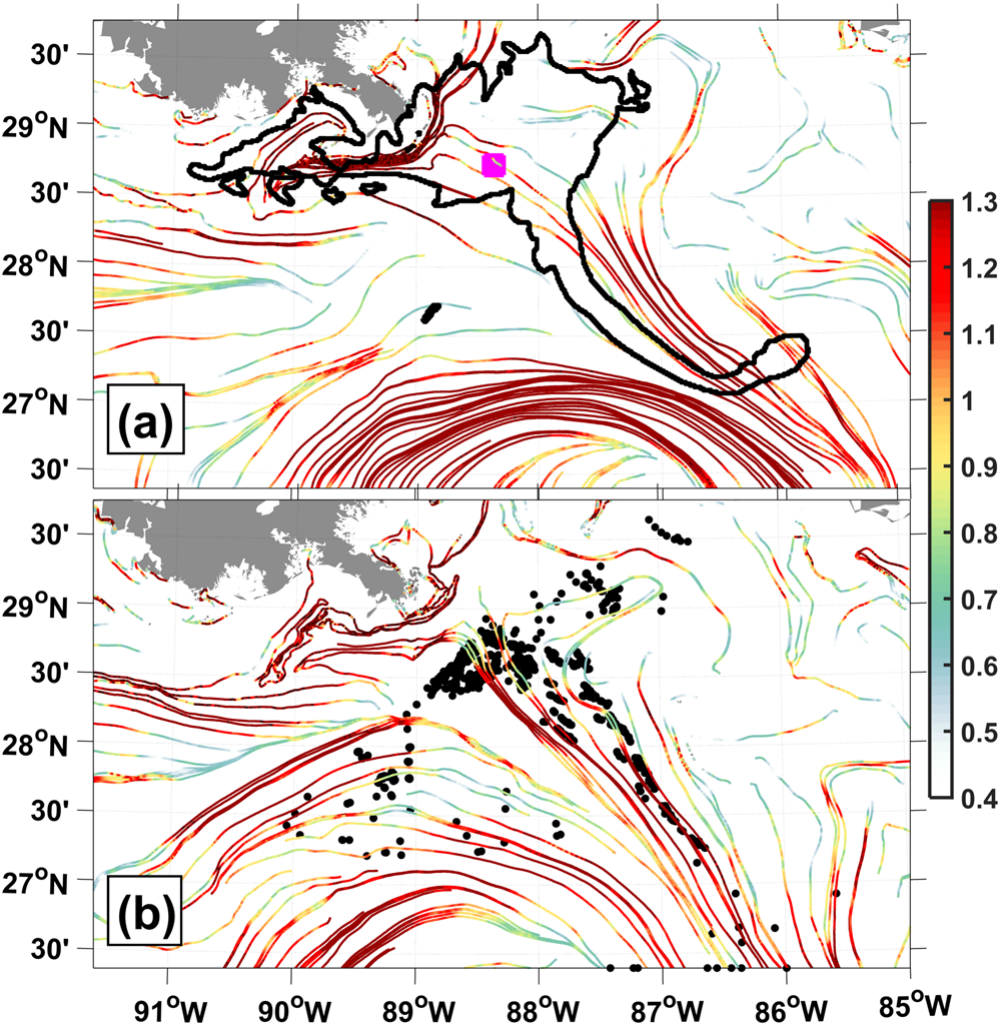
Climatological LCSs colored according to their climatological attraction strength lnc*ρ* for (**a**) May and (**b**) July in the northern GoM. Superimposed in (**a**) is the oil outline (black) from the Deepwater Horizon spill as seen on May 17, 2010; the Macondo well location (magenta square) is also shown. In (**b**), black dots are the daily positions of GLAD drifters from July 29 to August 2, 2012. Created with Matlab R2016B (www.mathworks.com).

During the DwH spill in May 2010, a current resembling a localized jet was responsible for a significant redistribution of the sea-surface oil slick. The resulting prominent filament became known as a “tiger tail”^50^. The tiger tail stretched along the direction indicated by the cLCSs for May (Fig. 3a). We also note that between 28.5–29.25°N and 89.5–90.5°W, the oil outline closely conforms to the cLCSs just south and west of the Mississippi delta (Fig. 3a).

For the most part of July 2012, a filament similar to the tiger tail was observed with GLAD drifters, sea-surface temperature and chlorophyll^8^; July’s cLCSs accurately show the direction of stretching (Fig. 3b).

#### Ixtoc oil spill

**Figure 4:**
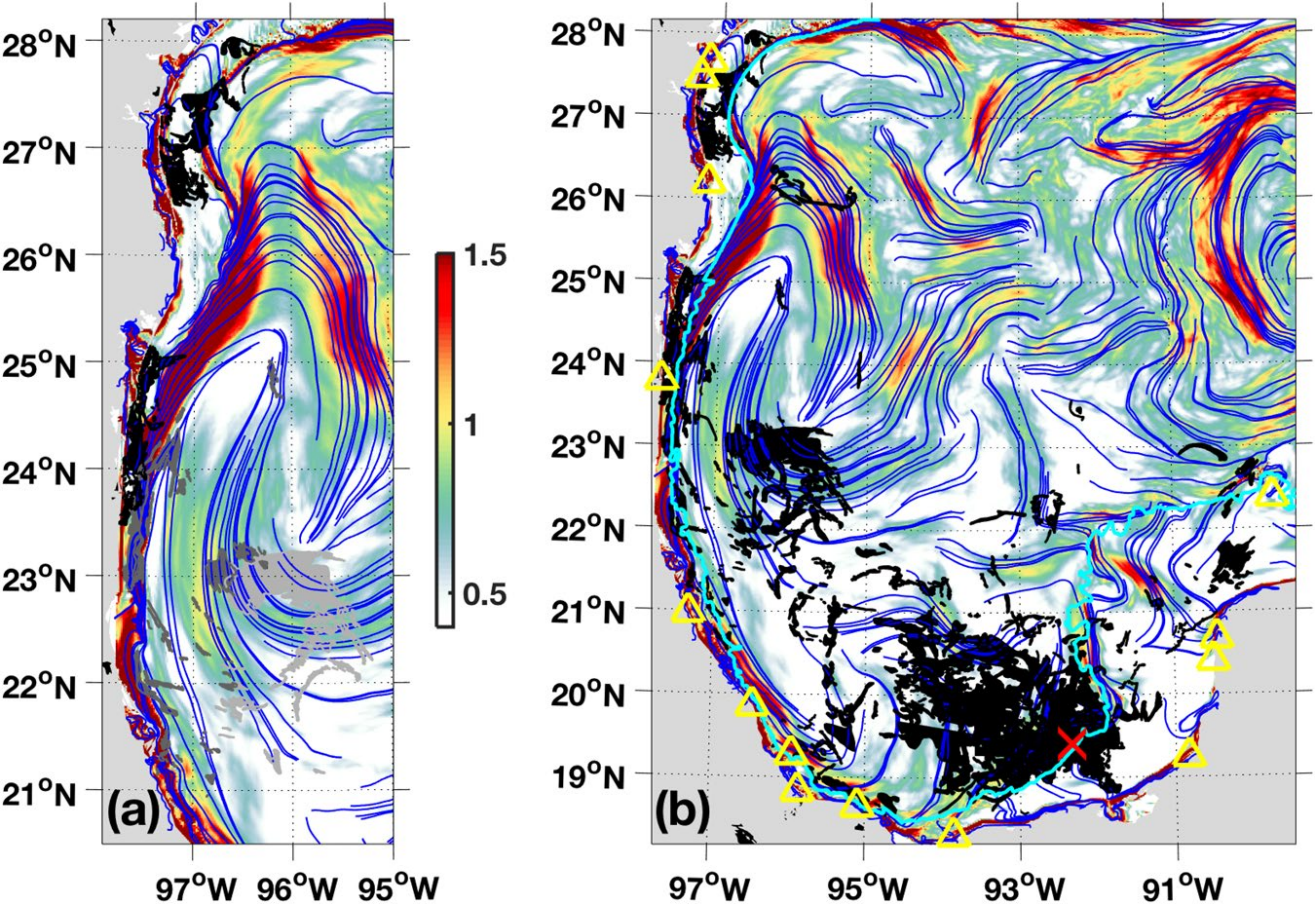
Climatological LCSs (blue curves) on climatological attraction strength (lnc*ρ*; colors) for August in the western GoM. (**a**) Superimposed in the left panel is oil from the *Ixtoc* spill as observed (Landsat/MSS satellite sensor) on August 1 (light gray), August 2 (gray) and August 21 (black), 1979. (**b**) Superimposed in the right panel is oil (black) from the *Ixtoc* spill as observed (Landsat/MSS and CZCS satellite sensors) through the almost 10-month duration of the blowout. The location of the blowout (92.33°W,19.41°N) is marked with a red cross and the 50-m isobath is plotted in cyan. Confirmed beachings reported by Sun *et al*.^29^ are marked with yellow triangles. Created with Matlab R2016B (www.mathworks.com).

Similar to the DwH accident, the *Ixtoc* rig off the coast of Mexico exploded in June 1979 (the blowout location is marked in Fig. 4b). The well was capped in March 1980, spilling the second largest accidental oil release after the DwH incident. Oil trajectories had two salient characteristics in that event: firstly the Yucatan shelf just east of the well remained relatively isolated; and secondly, the oil with the longest trajectories moved north and west, impacting the western GoM coast^29^. We find these patterns in the cLCS fields for trajectories originating in the vicinity of the accident. And again, this is consistent with the northward advection along the western margin that was reported by Zavala-Sanson *et al*.^33^, as a dominant dispersion scenario for point sources near the Ixtoc blowout.

Oil moving northwestward was documented in a two-day sequence of satellite images starting on August 1, 1979 (Fig. 4a). On the first day, oil is positioned over August’s cLCSs that direct westward and then northward at about 95–97°W, 22–23°N. The satellite image next day (August 2) shows that oil moved westward and northward following these cLCSs; note that not much filamentation is observed, possibly due to cloud coverage. In this second-day image, oil can also be seen closer to the coast, along 300 km of highly-attractive cLCSs that are aligned with the 50-m isobath (the isobath is shown in Fig. 4b). However, it seems unlikely that oil stretching along cLCSs over the the 50-m isobath on August 2, made it from the oil observed on the previous day at about 23°N–it would have required a velocity of one to two meters per second towards the coast. This suggests that on August 1, oil was already closer to the coast, but covered by clouds. The next available image with Ixtoc oil is on August 29, 1979. In this image, oil can be seen even closer to the coast at about 23.5–25°N and 26.5–28°N, near highly-attractive coastal cLCSs (which can also be seen in Fig. 2). These coastal cLCSs with with c *ρ* maxima, are consistent with the coastal vulnerability (attraction of synthetic drifters towards the coastline) reported by Thyng & Hetland^49^, as mentioned above.

When all the available satellite observations from the Ixtoc spill are plotted together, the 50-m isobath of the Yucatan shelf emerges as an effective transport barrier, although with some exceptions. The spill originated just off the 50-m isobath less than 100 km west of the Yucatan shelf. However, only a small amount of oil–relative to the amount to the north, west and northwest of the blowout–moved eastward onto the Yucatan shelf (Fig. 4b).

Oil moved southeast from the blowout towards highly-attractive coastal cLCSs near (91.8°W,18.7°N), along weakly-attracting cLCSs; thus cLCSs capture the most significant example of cross-shelf transport in these data. With one exception, all confirmed beachings along the Mexican and U.S. coasts happened where near-coast cLCSs have c *ρ* maxima (Fig. 4b; see also Fig. 2).

### Time-variability of the transport patterns

In this subsection we make a few remarks regarding the temporal variability of the transport patterns we have described above. Temporal variability behind cLCSs can be assessed by superimposing the LCSs from the twelve dynamical systems over which the CG-tensor averaging takes place. In the Supporting Information (Appendix C) we show quantitatively that the cLCSs are effectively equivalent to the superposition of LCSs from the dynamical systems in the averaging period. The superposition of LCSs confirm the transport patterns described above.

The similarity between a month’s cLCSs and the superposition of LCSs from 7-day flow maps spanning that month, suggests that the climatological velocity does not have much temporal variability over 30-day periods. However, in Appendix D of the Supporting Information, we show that our method cannot be simplified further by first monthly-averaging the climatological velocity and then computing streamlines.

It is when a suitable low-pass filter has been applied to the velocity field that recurrent, coherent pathways may emerge. When using the instantaneous velocity, the LCSs from the different dynamical systems have comparable stretching strengths, but orientation is more varied, and the cLCSs resemble a disorganized collection of LCSs.

There are several characteristics that confirm the quasi-steady nature of our results. The spatial location and strength of the LCS from different 7-day dynamical systems spanning a month are very similar to the corresponding cLCSs. One cLCS map per month is enough to anticipate a range of known circulation patterns. Several of the cLCS maps are qualitatively similar from month to month. Indeed, Gough *et al*.^36^ found good agreement between simulated and observed transport patterns, and cLCSs computed from yearly-averaging 7-day CG tensors.

## Concluding remarks

We have presented several independent confirmations that the cLCSs extract kinematics from multi-year time-series, synthesizing important information. cLCSs strongly constrain the ensemble-mean transport sustained by the instantaneous circulation, thus linking the climatological and instantaneous velocity fields. To the best of our knowledge, this is the first time that a velocity climatology has been shown to preserve meaningful transport information from the instantaneous velocity. Our results agree with data beyond the time period spanned by our velocity climatology, revealing that persistent, or recurrent, circulation has been found. Additionally, known prominent transport in the GoM are accurately depicted, including some of the highest-profile circulation patterns observed in recent history. These characteristics suggest the utility of our Lagrangian transport climatology for a variety of applications. As examples, those planning for environmental-pollution prevention and response may use the monthly cLCS maps to identify regions at high risk of being visited by contaminants, depending on the location of a pollution source. Regions that are isolated or stagnated are unlikely to be impacted if the pollution source lies outside, but will be heavily impacted if the source is within. Likely transport patterns can also be deduced, e.g., the tiger-tail filaments in the vicinity of the DwH. Our method generalizes the commonly used approach of running multiple simulations to compute the probability of contact given a point source. This is because cLCS maps classify regions’ risk of contact given a generic oil spill: Our results do not require, and therefore do not depend on, knowing the source’s location *a priori*. At the same time, our method provides more information relative to the probability-of-contact approach, because it clearly depicts likely pathways. These types of risk maps provide valuable information complementing oil-spill forecasts (e.g., Barker^39^). We emphasize that, by definition, a climatology discards weather patterns. Consequently, our results are a complement, not a substitute, for instantaneous-velocity simulations.

Other applications for the cLCS method may arise from recognizing the persistent or recurrent nature of kinematics as seen through the cLCSs method, for instance, for determining ideal navigation routes in a climatological sense, or possibly by gaining insight into the nature of the dynamics responsible for the low-frequency kinematics depicted by cLCSs.

Our work shows that it is possible to find quasi-steady, general patterns that describe important aspects of the inherently time-dependent, chaotic problem of oceanic Lagrangian transport—this appraises new applications as practical.

## Electronic supplementary material

Supporting Information

## References

[1] LaCasce JH (2008). Statistics from Lagrangian observations. Progress in Oceanography.

[2] Haller G, Yuan G (2000). Lagrangian coherent structures and mixing in two-dimensional turbulence. Physica D.

[3] Samelson RM (2013). Lagrangian motion, coherent structures, and lines of persistent material strain. Annual Review of Marine Science.

[4] Haller, G. Lagrangian Coherent Structures. *Annual Review of Fluid Mechanics* **47**, 140906185740003 10.1146/annurev-fluid-010313-141322 (2015).

[5] Haller G, Beron-Vera FJ (2012). Geodesic theory of transport barriers in two-dimensional flows. Physica D.

[6] Farazmand M, Blazevski D, Haller G (2014). Shearless transport barriers in unsteady two-dimensional flows and maps. Physica D.

[7] Haller G (2011). A variational theory of hyperbolic Lagrangian Coherent Structures. Physica D.

[8] Olascoaga MJ (2013). Drifter motion in the Gulf of Mexico constrained by altimetric Lagrangian Coherent Structures. Geophys. Res. Lett..

[9] Beron-Vera FJ (2015). Dissipative inertial transport patterns near coherent Lagrangian eddies in the ocean. Chaos.

[10] Bleck R (2002). An oceanic general circulation model framed in hybrid isopycnic-Cartesian coordinates. Ocean Modell..

[11] Cummings JA (2005). Operational multivariate ocean data assimilation. Q. J. Royal Meteorol. Soc..

[12] Cummings, J. A. & Smedstad, O. M. Variational data analysis for the global ocean. In Park, S. K. & Xu, L. (eds) *Data Assimilation for Atmospheric, Oceanic and Hydrologic Applications*, vol. 2, chap. 13, 10.1007/978-3-642-35088-7-13 (Springer-Verlag Berlin Heidelberg 2013).

[13] Beron-Vera FJ, LaCasce JH (2016). Statistics of simulated and observed pair separations in the Gulf of Mexico. J. Phys. Oceanogr..

[14] Beron-Vera FJ, Olascoaga MJ (2009). An assessment of the importance of chaotic stirring and turbulent mixing on the West Florida Shelf. J. Phys. Oceanogr..

[15] Keating SR, Smith KS, Kramer PR (2011). Diagnosing lateral mixing in the upper ocean with virtual tracers: Spatial and temporal resolution dependence. J. Phys. Oceanogr..

[16] Shulman I, Ramp SR, Anderson S, Metzger EJ, Sakalaukus P (2013). Impact of remote forcing, model resolution and bathymetry on predictions of currents on the shelf. Dynamics of Atmospheres and Oceans.

[17] Sandery PA, Sakov P (2017). Ocean forecasting of mesoscale features can deteriorate by increasing model resolution towards the submesoscale. Nature Comm..

[18] Soufflet Y (2016). On effective resolution in ocean models. Ocean Modelling.

[19] National Oceanic and Atmospheric Administration & U.S. Coast Guard. *Characteristics of Response Strategies: A Guide for Spill Response Planning in Marine Environments*. A joint publication of the American Petroleum Institute, the National Oceanic and Atmospheric Administration, the U.S. Coast Guard and the U.S. Environmental Protection Agency. https://response.restoration.noaa.gov/sites/default/files/Characteristics_Response_Strategies.pdf (2010).

[20] Melsom A, Counillon F, LaCasce JH, Bertino L (2012). Forecasting search areas using ensemble ocean circulation modeling. Ocean Dynamics.

[21] Chen C (2012). FVCOM model estimate of the location of Air France 447. Ocean Dynamics.

[22] Lalli, C. M. & Parsons, T. R. *BIOLOGICAL OCEANOGRAPHY: AN INTRODUCTION* (Pergamon Press Ltd. 1993).

[23] Miller, C. *BIOLOGICAL OCEANOGRAPHY* (Blackwell Publishing 2004).

[24] Hadjighasem A, Farazmand M, Haller G (2013). Detecting invariant manifolds, attractors, and generalized KAM tori in aperiodically forced mechanical systems. Nonlinear Dyn..

[25] Onu K, Huhn F, Haller G (2015). LCS Tool: A computational platform for Lagrangian coherent structures. J. Comp. Sci..

[26] Poje AC (2014). The nature of surface dispersion near the Deepwater Horizon oil spill. Proc. Nat. Acad. Sci. USA.

[27] Jacobs GA (2014). Data assimilation considerations for improved ocean predictability during the Gulf of Mexico Grand Lagrangian Deployment (GLAD). Ocean Modell..

[28] Coelho EF (2015). Ocean current estimation using a Multi-Model Ensemble Kalman Filter during the Grand Lagrangian Deployment experiment (GLAD). Ocean Modell..

[29] Sun S, Hu C, Tunnell JW (2015). Surface oil footprint and trajectory of the Ixtoc-I oil spill determined from Landsat/MSS and CZCS observations. Marine Pollution Bulletin.

[30] Froyland G, Padberg K, England MH, Treguier AM (2007). Detection of coherent oceanic structures via transfer operators. Phys. Rev. Lett..

[31] Vukovich FM (2007). Climatology of Ocean Features in the Gulf of Mexico Using Satellite Remote Sensing Data. Journal of Physical Oceanography.

[32] Lindo-Atichati D, Bringas F, Goni G (2013). Loop Current excursions and ring detachments during 1993–2009. International Journal of Remote Sensing.

[33] Zavala-Sansón L, Pérez-Brunius P, Sheinbaum J (2017). Point source dispersion of surface drifters in the southern Gulf of Mexico. Environmental Research Letters.

[34] Martnez-López B, Zavala-Hidalgo J (2009). Seasonal and interannual variability of cross-shelf transports of chlorophyll in the Gulf of Mexico. Journal of Marine Systems.

[35] Zhang Z, Hetland R (2012). A numerical study on convergence of alongshore flows over the Texas-Louisiana shelf. Journal of Geophysical Research.

[36] Gough, M. *et al*. Persistent Lagrangian transport patterns in the northwestern Gulf of Mexico. *Submitted JPO*. https://arxiv.org/abs/1710.04027 (2017).

[37] Le Henaff M (2012). Surface Evolution of the Deepwater Horizon Oil Spill Patch: Combined Effects of Circulation and Wind-Induced Drift. Environmental Science and Technology.

[38] Weisberg RH, Lianyuan Z, Liu Y (2017). On the movement of Deepwater Horizon Oil to northern Gulf beaches. Ocean Modelling.

[39] Barker CH (2011). A statistical outlook for the Deepwater Horizon oil spill. In Monitoring and Modeling the Deepwater Horizon Oil Spill: A Record-Breaking Enterprise.

[40] Ji ZG, Johnson WR, Li Z (2011). Oil spill risk analysis model and its application to the Deepwater Horizon oil spill using historical current and wind data. In Monitoring and Modeling the Deepwater Horizon Oil Spill: A Record-Breaking Enterprise.

[41] Tulloch, R., Hill, C. & Jahn, O. Possible Spreadings of Buoyant Plumes and Local Coastline Sensitivities Using Flow Syntheses From 1992 to 2007. In *Monitoring and Modeling the Deepwater Horizon Oil Spill: A Record Breaking Enterprise*, 245–255 10.1029/2011GM001125 (2011).

[42] Brink K (2016). Cross-Shelf Exchange. Annual Review of Marine Science.

[43] Yang H, Weisberg RH, Niiler PP, Sturges W, Johnson W (1999). Lagrangian circulation and forbidden zone on the West Florida Shelf. Cont. Shelf. Res..

[44] Olascoaga MJ (2006). Persistent transport barrier on the West Florida Shelf. Geophys. Res. Lett..

[45] Olascoaga MJ (2010). Isolation on the West Florida Shelf with implications for red tides and pollutant dispersal in the Gulf of Mexico. Nonlin. Proc. Geophys..

[46] Sturges, W., Niiler, P. P. & Weisberg, R. H. Northeastern Gulf of Mexico Inner Shelf Circulation Study. *OCS Report MMS. U.S. Minerals Management Service* **Final Report**, 35–1 (2001).

[47] Li Z, Weisberg RH (1999). West Florida shelf response to upwelling favorable wind forcing 1: Kinematics. Journal of Geophysical Research.

[48] Li Z, Weisberg RH (1999). West Florida continental shelf response to upwelling favorable wind forcing 2. Dynamics. Journal of Geophysical Research.

[49] Thyng, K. M. & Hetland, R. D. Texas and Louisiana coastal vulnerability and shelf connectivity. *Marine Pollution Bulletin*, 10.1016/j.marpolbul.2016.12.074 (2017). 10.1016/j.marpolbul.2016.12.074 28081958

[50] Olascoaga MJ, Haller G (2012). Forecasting sudden changes in environmental pollution patterns. Proceedings of the National Academy of Sciences of the United States of America.

