# Supplementary material for: Extracting quasi-steady Lagrangian transport patterns from the ocean circulation: An application to the Gulf of Mexico
Source: Sci Rep. 2018 Mar 26;8:5218. doi: 10.1038/s41598-018-23121-y (PMC5980098; doi:10.1038/s41598-018-23121-y)
Supplement: Supplementary file 1 — Supporting Information [file 41598_2018_23121_MOESM1_ESM.pdf]

Supporting Information for "Extracting quasi-steady Lagrangian transport patterns from the ocean circulation: An application to the Gulf of Mexico"

R. Duran[1,*], F. J. Beron-Vera[2], and M. J. Olascoaga[3]

[1]College of Earth Ocean and Atmospheric Sciences, Oregon State University, Corvallis, Oregon, USA
[*]Corresponding author: rduran@ceoas.oregonstate.edu
[2]Department of Atmospheric Sciences, Rosenstiel School of Marine and Atmospheric Science, University of Miami, Miami, Florida, USA
[3]Department of Ocean Sciences, Rosenstiel School of Marine and Atmospheric Science, University of Miami, Miami, Florida, USA

Contents

- Appendix A. Divergence of the climatological velocity.
- Appendix B. Resolution of the cLCSs grid.
- Appendix C. A quantitative comparison between cLCSs and LCSs.
- Appendix D. Comparing cLCSs with the streamlines for the monthly-averaged climatological velocity.

Divergence of the climatological velocity

In this section we investigate if the cLCSs' attraction is due to confluence (i.e. divergence-free attraction) or convergence (attraction with negative divergence). We use the notation described in the paper. Let $\delta(\mathbf{x},t) := \nabla \cdot \mathbf{v}(\mathbf{x},t)$ be the Eulerian divergence of our two-dimensional climatological velocity field (described in the paper). Daily values of the Eulerian divergence from our climatology have several persistent features that are aptly captured in the yearly mean: These characteristic features include strong positive divergence with negative divergence next to it along the western boundary of the GoM, positive divergence between 92-96 °W at about 19°N, and the LC is characterized by positive divergence to both sides of negative divergence (Fig. 1).

We are interested in the divergence along the paths we used for our cLCSs computations, to understand if the

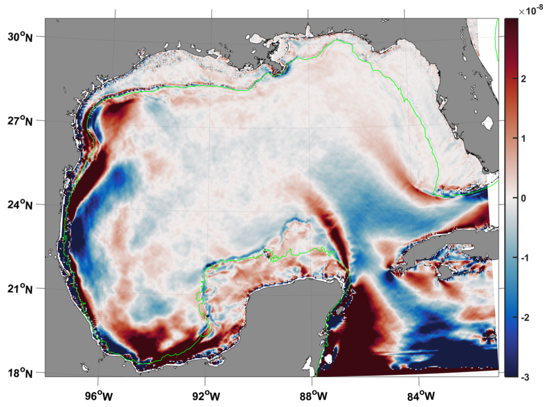

Figure 1. Yearly average of the Eulerian divergence (day^{-1}) computed from the daily values in our climatological velocity (described in the paper). The 50-m isobath is shown in green. Saturated colors are an order of magnitude bigger than the shown colorscale. Created with Matlab R2016B (www.mathworks.com).

back-in-time repulsion we compute can be associated with an increase in sea-surface area due to positive divergence. In forward time this would imply that the attraction we report is associated with an area decrease due to negative divergence. We can use the equation for the change of a material area $\mathrm{d}A(t)/\mathrm{d}t = \delta(t)A(t)$ which has the solution

$$A(t) = A_0 \exp\left(\int_{t_0}^{t} \delta\left(\mathbf{F}_{t_0}^{t'}(\mathbf{x}_0), t'\right) \mathrm{d}t'\right) \tag{1}$$

Define the parameter

$$\alpha := A(t)/A_0 \tag{2}$$

(These equations are often treated in textbooks of classical mechanics under Liouville's theorem and subsequent lemmas.) We compute α by evaluating the exponential in (1) using the same flow maps $\mathbf{F}_{t_0}^{t}(\mathbf{x}_0)$ that we used for the computation of cLCSs (described in the paper). Note that the fractional change of area is the Jacobian determinant of the transformation $\mathbf{F}_{t_0}^{t}$, i.e. $\alpha = \det\left(\mathrm{D}\mathbf{F}_{t_0}^{t}\right)$. When $\alpha \approx 1$, shrinking or expanding of areas is negligible over the integration period and attraction (in forward time) would be due to confluence.

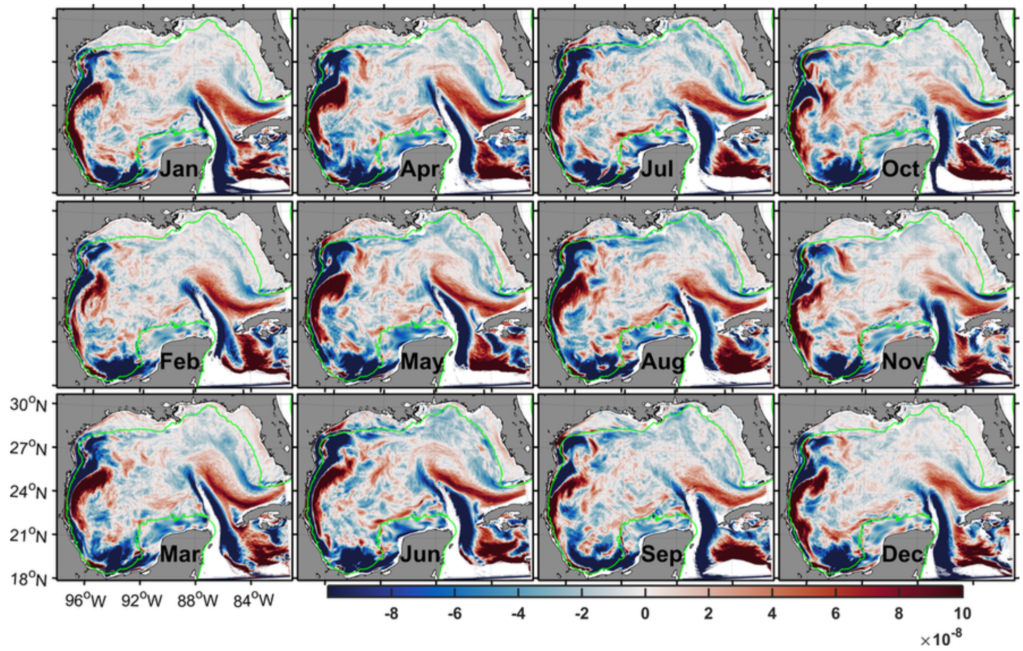

Figure 2. Monthly-averages of the parameter α defined in (2), and using the same flow maps as in the computation of cLCSs. The colorscale represents the signed distance from one, e.g., a value of -2×10^{-8} represents $1 - 2 \times 10^{-8} = 0.99999998$. The monthly averages are computed by averaging the values of α from each of the twelve dynamical systems in a month (as described in the paper). α is plotted as a function of the trajectories' initial position $\mathbf{x}_0$ used to compute the exponential in (1). The 50-m isobath is shown in green. Saturated colors are an order of magnitude bigger than the shown colorscale. Created with Matlab R2016B (www.mathworks.com).

We compute monthly-mean fields by averaging the twelve α fields, obtained from the flow maps of the twelve dynamical systems we used to characterize each month (Fig. 2). A closer look at the values of α for each month is presented in table 1. Even the smallest (0.9995) and biggest values (1.00005) of α throughout our climatology, result in negligible changes of area. The absolute maximum reduction of area as it was advected with the flow map corresponds to 0.05% of the original area; the absolute maximum increase is 0.005% of the original area. Furthermore, most values of α are much closer to one than these extreme values, as can be seen in the probability distributions (Fig. 3). We note that distributions are skewed towards negative values (Table 1 and Fig. 3).

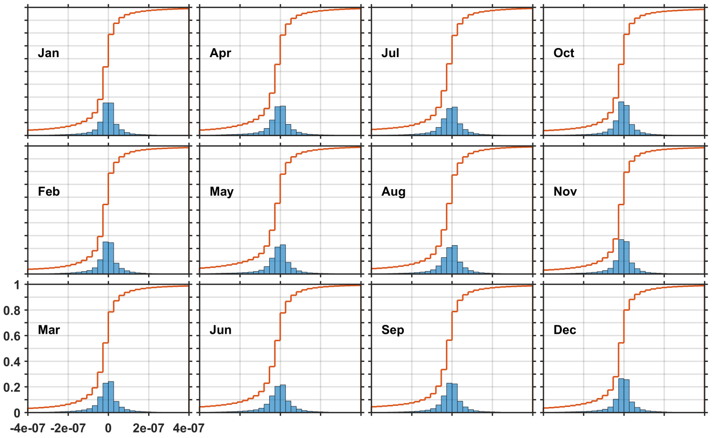

Figure 3. Probability distribution (blue bars, abscissa) and cumulative probability distribution (orange lines, abscissa) for all the values of α (defined in (2) and computed using the same flow maps with which cLCSs were computed) in each month, as a function of the signed distance from one (ordinate; e.g., a value of -1×10^{-7} represents $1-1\times10^{-7}=0.9999999$). Created with Matlab R2016B (www.mathworks.com).

In these back-in-time integrations, negative values work to impede back-in-time repulsion and therefore forward-in-time attraction. Thus, we conclude that the effect of divergence is negligible, and that out of the negligible effect, the most significant part acts to counter the attraction we report.

Month	Mean	StdDev	AbsMin	1stQuartile	Median	3rdQuartile	AbsMax
Jan	-3e-07	5e-06	-3e-04	-3e-08	-3e-09	2e-08	4e-06
Feb	-3e-07	5e-06	-3e-04	-3e-08	-3e-09	2e-08	5e-06
Mar	-4e-07	7e-06	-3e-04	-4e-08	-4e-09	2e-08	5e-06
Apr	-4e-07	7e-06	-5e-04	-4e-08	-5e-09	2e-08	1e-05
May	-4e-07	7e-06	-5e-04	-4e-08	-6e-09	2e-08	6e-06
Jun	-5e-07	8e-06	-5e-04	-5e-08	-7e-09	2e-08	4e-06
Jul	-4e-07	6e-06	-4e-04	-5e-08	-7e-09	2e-08	3e-06
Aug	-4e-07	7e-06	-4e-04	-4e-08	-6e-09	2e-08	7e-06
Sep	-4e-07	6e-06	-4e-04	-4e-08	-6e-09	2e-08	5e-05
Oct	-3e-07	5e-06	-2e-04	-3e-08	-5e-09	2e-08	2e-05
Nov	-2e-07	4e-06	-3e-04	-3e-08	-3e-09	2e-08	5e-05
Dec	-2e-07	4e-06	-3e-04	-3e-08	-3e-09	2e-08	6e-06

Table 1. Statistics for all the values of α spanning the twelve dynamical systems in each month; values are presented as the signed distance from one, e.g. -3e-07 represents a value of 1-3e-07=0.9999997.

Resolution of the cLCSs grid

In this section we show that cLCSs remain the same when computed with twice the resolution, i.e. with a computational grid that has $dx = dy \approx 800$m, rather than 1.7km. To this purpose, May is presented as an example (Fig. 4), showing that cLCSs remain the same with either resolution. This result does not depend on the choice of month.

A quantitative comparison between cLCSs and LCSs

cLCSs are the squeezelines of a monthly-mean Cauchy-Green (CG) tensor; the averaging is described in the paper for which this is a supplement. Here we show that the LCSs from the twelve CG tensors over which we average,

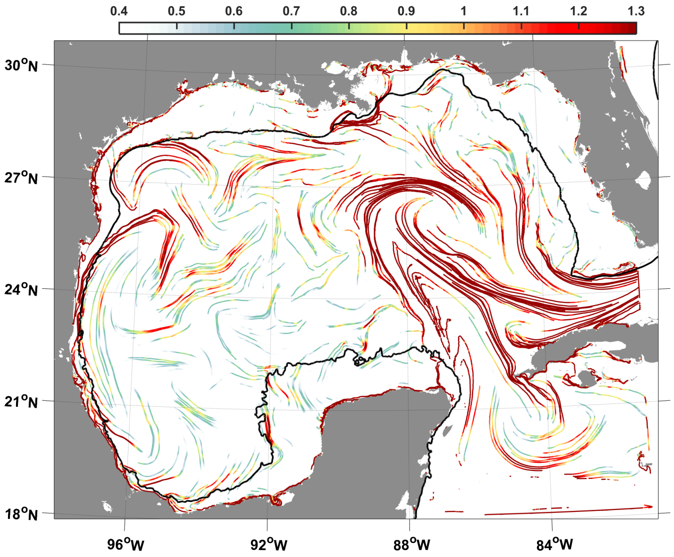

Figure 4. cLCSs for May computed with a computational grid that is double the resolution used in the paper. Color represents climatological attraction $\ln c\rho$. Created with Matlab R2016B (www.mathworks.com).

often persist in similar positions, and that the corresponding cLCSs are similarly located. Our approach is to compare the cLCSs to the superposition of LCSs from all of the CG tensors over which we average when computing the monthly-mean CG tensor. A visual inspection between the superposition of all LCSs (Fig. 5) readily suggests a striking similarity to the corresponding cLCSs (compare to Fig. 2 of the paper). For a quantitative analysis, LCSs and cLCSs are first interpolated to equidistant points along the curve, making them directly comparable. We then use a 0.175×0.175 degree grid to compute the probability that any given cell contains cLCS (x, y) points, and compare that to the probability that the same cell has LCS (x, y) points from any of the dynamical systems in the averaging period (Fig. 6). A robust least squares regression shows a significantly-correlated linear relation for the cell-wise comparison of the two probability distributions. The details for each month's linear regression are shown in table 2.

Month	Correlation	Slope
Jan	0.74	0.55
Feb	0.75	0.47
Mar	0.77	0.59
Apr	0.64	0.49
May	0.74	0.53
Jun	0.71	0.52
Jul	0.73	0.46
Aug	0.7	0.46
Sep	0.76	0.47
Oct	0.76	0.48
Nov	0.72	0.5
Dec	0.76	0.51

Table 2. Correlation between the cLCSs and LCSs probabilities for each month, values above 0.05 are significant with 95% confidence. The slope of the robust least-squares linear fit is also shown. All y-intercepts are between 5 and 7 $\times 10^{-5}$.

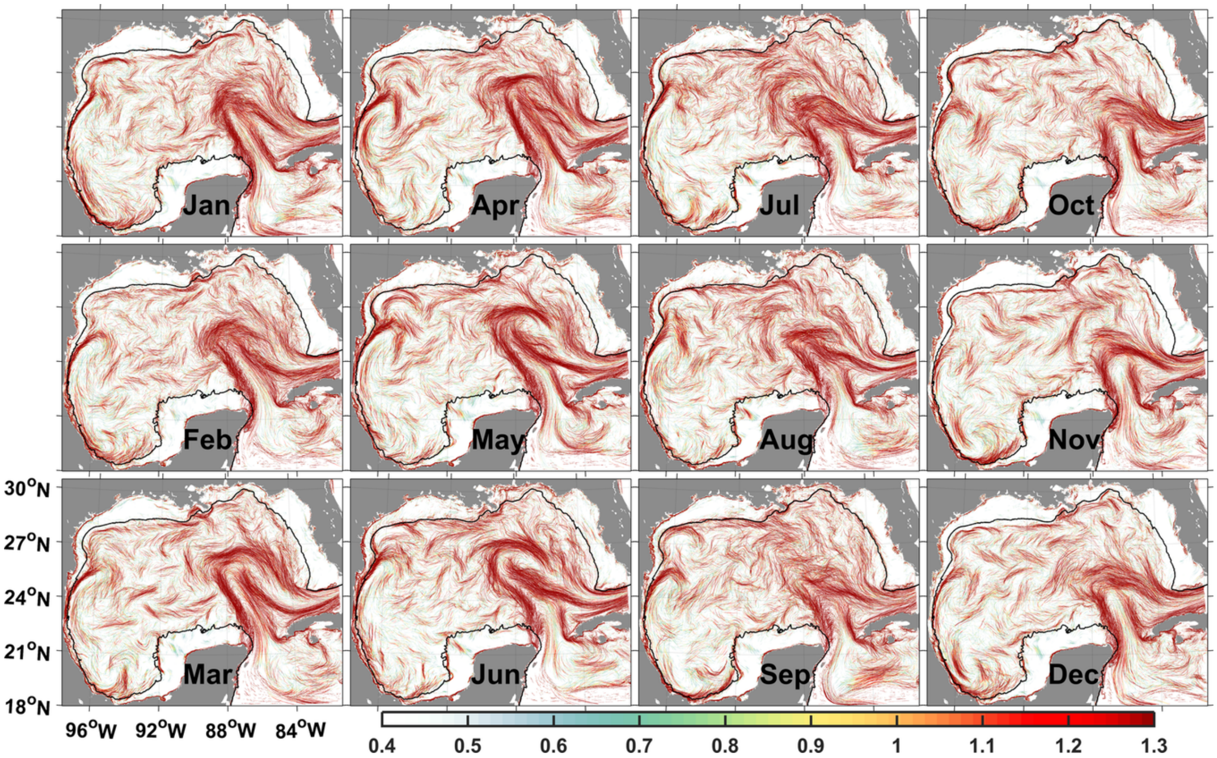

Figure 5. Superposition of the LCSs from the 12 dynamical systems used to compute the monthly-averaged Cauchy-Green tensor from where climatological LCS are computed (shown in Fig. 2 of the paper). Colors represents the LCSs' attraction strength $\ln\rho$. The 50-m isobath is indicated in black. Created with Matlab R2016B (www.mathworks.com).

Effectively, LCSs from the climatological velocity identify quasi-steady transport patterns over a month time period, and computing cLCSs is an efficient way of extracting these patterns.

Comparing cLCSs with the streamlines for the climatological velocity's monthly mean

In the previous section we showed that the climatological velocity does not have much variability within a month timescale in the sense that the LCSs from the different dynamical systems spanning any month are very similar to the corresponding cLCSs. The purpose of this section is to show that our analysis cannot be simplified further by computing streamlines of the monthly-averaged climatological velocity, instead of the cLCSs that we compute. For example, April's monthly-averaged climatological-velocity streamlines show cross-shelf transport in Florida's western and northwestern shelves (Fig. 7). In contrast, April's cLCSs and LCSs show an isolated West Florida Shelf (cf. Fig. 2 of the paper and Fig. 5 of this supplement), in agreement with previous observational and numerical studies (described in the paper). The Yucatan and La-Tex shelves also show unrealistic cross-shore transport. Other months also show spurious transport patterns. Furthermore, the streamlines do not identify regions of isolation or stagnation, which are accurately identified by regional minima of $c\rho$.

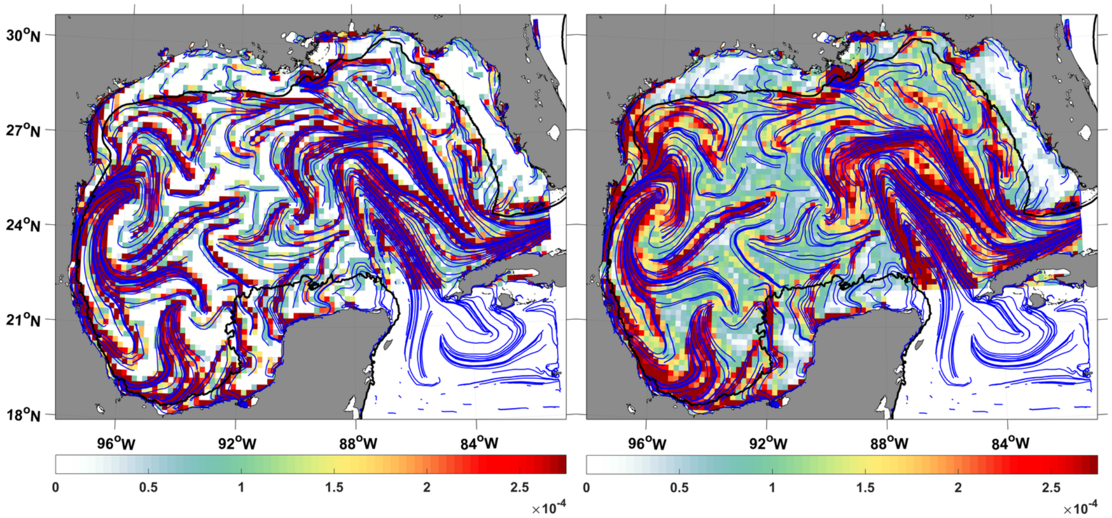

Figure 6. Both panels show the climatological Lagrangian Coherent Structures (cLCSs; blue lines) for April. In colors, the left panel shows the probability distribution (on a 0.175×0.175 degree grid) of April's cLCSs (x,y) positions. The right panel is the probability distribution (same grid) of the (x,y) positions of all the LCSs from each of the CG tensors that were averaged to get April's cLCSs. The 50-m isobath is shown in black. Plots for all months are qualitatively similar, therefore, only the comparison for the month with the smallest correlation is shown; correlations for all months are presented in table 2. Created with Matlab R2016B (www.mathworks.com).

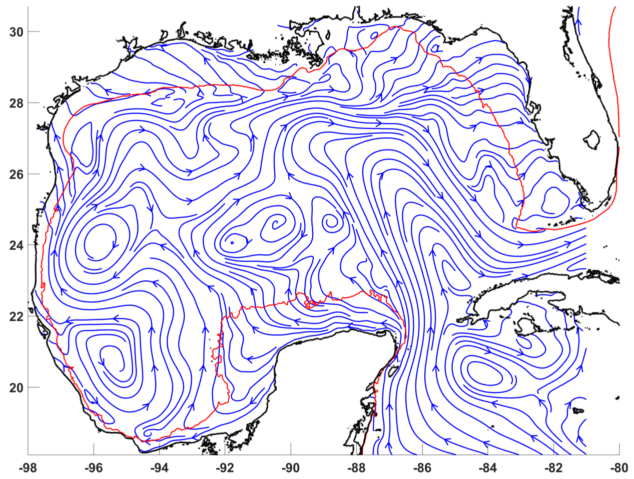

Figure 7. Streamlines (blue lines) for April computed from the climatological velocity that was used to compute April's cLCSs after monthly averaging; the 50-m isobath is shown in red. Notice the cross-shore flow throughout the West Florida and other shelves; compare to the cLCSs for April shown in Fig. 2 of the paper, and also the superposition of LCSs shown in Fig. 5 of this supplement. Created with Matlab R2016B (www.mathworks.com).
